# Thyrotoxicosis and Impending Thyroid Storm: A Rare Paraneoplastic Syndrome in an Infant With Hepatoblastoma

**DOI:** 10.1210/jcemcr/luad051

**Published:** 2023-06-15

**Authors:** Neha Vyas, Arino Neto, Mauri Carakushansky, Shilpa Gurnurkar

**Affiliations:** Division of Pediatric Endocrinology, Nemours Children's Health, Orlando, FL 32827, USA; Pediatrics Residency Program, Department of Pediatrics, Nemours Children's Health, Orlando, FL 32827, USA; Division of Pediatric Endocrinology, Nemours Children's Health, Orlando, FL 32827, USA; Division of Pediatric Endocrinology, Nemours Children's Health, Orlando, FL 32827, USA

**Keywords:** thyroid storm, hepatoblastoma, paraneoplastic syndrome

## Abstract

Graves’ disease is the most common cause of pediatric hyperthyroidism and thyrotoxicosis. Thyroid storm is a rare initial manifestation of Graves’ disease and represents an endocrine emergency. We report a case of transient hyperthyroidism, possibly a paraneoplastic syndrome presenting as impending thyroid storm in a patient with undiagnosed hepatoblastoma. To our knowledge, this is the first case of this association reported in children. A previously healthy 21-month-old male presented with abdominal pain and unremitting tachycardia. He was managed for thyrotoxicosis and impending thyroid storm. He subsequently was found to have hepatomegaly leading to a diagnosis of hepatoblastoma. Autoimmune markers for Graves’ disease were negative, along with a negative human chorionic gonadotropin. After initiation of neoadjuvant chemotherapy, he had complete resolution of thyrotoxicosis. Paraneoplastic syndromes may occur with any tumor. We present a unique case of a patient developing human chorionic gonadotropin-negative hyperthyroidism, possibly as a paraneoplastic syndrome from hepatoblastoma.

## Introduction

Graves’ disease (GD) is the most common cause of pediatric hyperthyroidism and thyrotoxicosis, accounting for about 96% of cases [1]. GD occurs from the formation of thyrotropin (TSH) receptor-stimulating antibodies to the thyrotropin receptor, leading to increased gland vascularity and excessive thyroid hormone production. Clinical manifestations can vary and include premature craniosynostosis in infancy; cardiovascular symptoms such as tachycardia and increased systolic blood pressure; menstrual irregularities such as oligomenorrhea or secondary amenorrhea; weight loss; and diarrhea [1].

Thyroid storm (TS) is a rare initial manifestation of GD and represents an endocrine emergency. In adults, it is estimated to occur in less than 1% of patients. In children, TS is an infrequent phenomenon with no documented incidence rate in the United States. Precipitating factors include cessation of antithyroid medication, surgical or trauma-related stress, or radioactive iodine therapy in a patient with underlying or poorly controlled GD [2]. Thyroid storm may lead to multisystem organ failure with manifestations of vomiting, diarrhea, tachycardia, fever, hepatic dysfunction, hypotension, and congestive heart failure [2].

We describe a rare presentation of human chorionic gonadotropin (HCG) negative thyrotoxicosis with impending thyroid storm in a 21-month-old male with a subsequent diagnosis of hepatoblastoma and propose that this could be a paraneoplastic syndrome.

As per our institutional review board (IRB) guidelines, case reports of 1 or 2 individuals do not require IRB review and approval; therefore, IRB review was not obtained. Written, informed consent was obtained from the patient's parent.

## Case Presentation

A previously healthy, 21-month-old Hispanic male presented with concerns of intermittent abdominal pain, diffuse abdominal tenderness, unremitting tachycardia, anxiety, bilateral exophthalmos, and a 3-month history of no weight gain. During the first 3 hours of hospitalization, the patient had a normal temperature (37.1 °C). His heart rate varied between 135 and 231 beats per minute, with normal respirations. Blood pressure was elevated at 128/73 mm Hg. Agitation and anxiety were present. During admission, no hepatosplenomegaly or abdominal masses were appreciated, though the examination was limited because of patient cooperation. An abdominal X-ray showed a moderate-to-large amount of stool, no organomegaly, mass effect, or abnormal calcification. His presentation was concerning for multiple differentials including, but not limited to, myocarditis, heart failure arrhythmias, pain, hyperthyroidism, or electrolyte abnormalities.

## Diagnostic Assessment

Initial laboratory evaluation confirmed thyrotoxicosis with a TSH <0.02 mcU/mL or mIU/L (reference range, 0.5-4.5 mcU/mL, or mIU/L), free T4 at 5.8 ng/dL (reference range, 0.8-2 ng/dL) or 74.6 pmol/L (reference range, 10.3-25.7 pmol/L), and free T3 at 15.4 pg/mL (reference range, 2.8-4.4 pg/mL) or 23.7 pmol/L (reference range, 4.3-6.7 pmol/L). Total T3 was not obtained with initial testing. His complete blood count included a white blood count of 14 × 10^9^/L (reference range, 7.73-13.12 × 10^9^/L), hemoglobin of 11.2 g/dL (reference range, 10.4-12.5 g/dL), platelets of 497 × 10^9^/L (reference range, 150-400 × 10^9^/L), and absolute neutrophil count of 6.9 K/UL (reference range, 2.47-6.41 K/UL). Liver enzymes also were obtained at alanine transaminase 92 U/L (reference range, 20-60 U/L) and alanine transaminase 24 U/L (reference range, 5-45 U/L). A thyroid ultrasound showed diffusely coarsened echotexture with diffuse hyperemia without focal nodules or enlarged cervical lymph nodes and borderline enlarged gland (right lobe 2.4 × 1.2 × 1.0 cm and left lobe 3.2 × 1.1 × 0.8 cm) [3].

## Treatment

He was treated with methimazole 2.5 mg 3 times daily (0.75 mg/kg/d), potassium iodide 50 mg 3 times daily, and propranolol 7 mg 3 times daily (2 mg/kg/d). After 3 days of therapy, laboratory improvement was noted with TSH <0.02 mcU/mL or mIU/L (reference range, 0.5-4.5 mcU/mL), free T4 at 2.3 ng/dL (reference range, 0.8-2 ng/dL) or 29.6 pmol/L (reference range, 10.3-25.7 pmol/L), and total T3 at 194 ng/dL (reference range, 92-248 ng/dL) or 2.9 nmol/L (reference range, 1.3-3.8 nmol/L).

He was discharged home in stable condition on methimazole and propranolol. He completed a 10-day course of propranolol. Interestingly, all thyroid autoantibodies were negative, including thyroid stimulating immunoglobulin, thyrotropin receptor antibody (TRAb), anti-thyroglobulin, and anti-thyroid peroxidase antibodies. Three weeks after his initial hospital admission, he developed a diffuse urticarial rash on his face, arms, and legs. Differential diagnoses included viral exanthem and allergic reactions. Because of concern for allergic reaction to methimazole, it was stopped, and propranolol restarted at 2 mg/kg/d. Four days after the onset of the rash, he developed persistent fevers and was readmitted. On readmission, his thyroid studies were altered, including TSH <0.02 mcU/mL or mIU/L (reference range, 0.5-4.5 mcU/mL or mIU/L), free T4 at 5.3 ng/dL (reference range, 0.8-2 ng/dL) or 68.2 pmol/L (reference range, 10.3-25.7 pmol/L), and free T3 at 11.3 pg/mL (reference range, 2.8-4.4 pg.mL) or 17.4 pmol/L (reference range, 4.3-6.7 pmol/L). His complete blood count included a white blood count of 17.6 × 10^9^/L (reference range, 7.73-13.12 × 10^9^/L), hemoglobin of 9.1 g/dL (reference range, 10.4-12.5 g/dL), platelets of 764 × 10^9^/L (reference range, 150-400 × 10^9^/L), and absolute neutrophil count of 9.5 K/UL (reference range, 2.47-6.41 K/UL). Liver enzymes were within normal limits (aspartate transaminase of 58 U/L [reference range, 20-60 U/L] and alanine transaminase of 9 U/L [reference range, 5-45 U/L]). Methimazole 2.5 mg 3 times per day (0.75 mg/kg/d) was restarted during the hospital stay without developing a rash.

## Outcome and Follow-up

**Figure 1.**
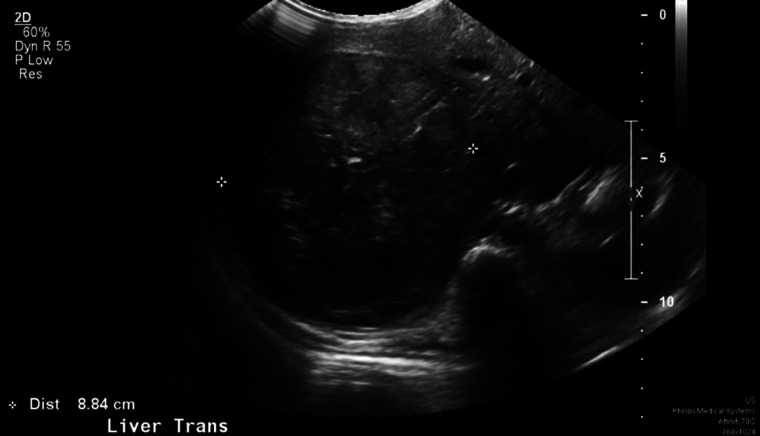
An ultrasound of the patient's abdomen showing a well-circumscribed, 11 × 10 × 10 cm solid mass occupying the right hepatic lobe.

**Figure 2.**
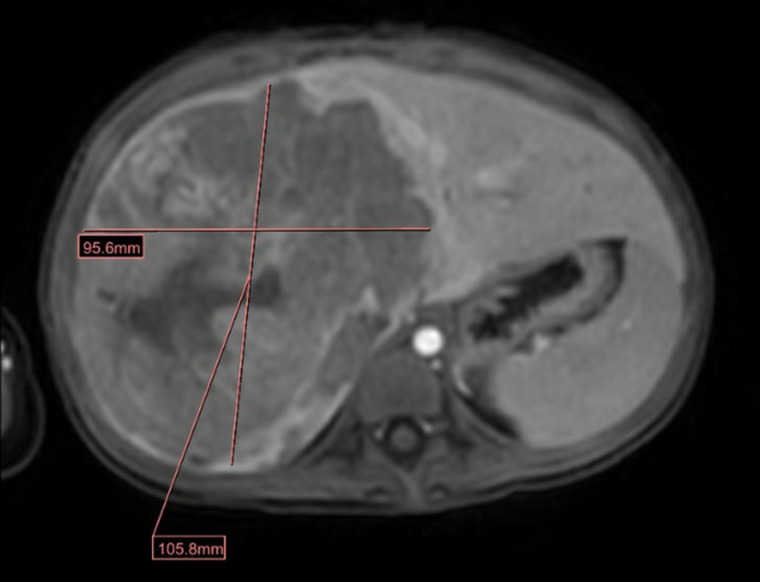
A magnetic resonance image (MRI) of the patient's abdomen showing a very large tumor (12.2 × 10.6 × 9.6 cm) in the right hepatic lobe, primarily involving the posterior segment.

Two weeks following his second discharge, the patient developed fevers, abdominal pain, diffuse waxing and waning rash, decreased activity, and reduced appetite. He was seen by his pediatrician who noted hepatomegaly (about 6 weeks after initial hospital admission). Abdominal computed tomography and ultrasound (Fig. 1) revealed a liver mass (11 × 10 × 10 cm) and a 7-mm peripherally placed pulmonary nodule in the left lower lobe. Subsequently, an abdominal magnetic resonance imaging scan (Fig. 2) confirmed the lesion in the right hepatic lobe (12.2 × 10.6 × 9.6 cm). The patient was admitted to the hematology-oncology department, and evaluation revealed an elevated alpha-fetoprotein level of 43 051 ng/mL (reference range, <6 ng/mL) or 35 568 IU/mL (reference range, <6 ng/mL), concerning for a hepatoblastoma which a tissue biopsy confirmed. HCG was sent to assess for markers of ectopic hormone production; however, levels were normal at 0.6 IU/L or 0.6 mIU/mL (reference range, <1.4 IU/L). He was initiated on neoadjuvant chemotherapy and methimazole was discontinued 1 week later because of laboratory findings of biochemical hypothyroidism with low free T4 at 0.7 ng/dL (reference range, 0.8-2 ng/dL) or 9 pmol/L (reference range, 10.3-25.7 pmol/L), and low total T3 at 66 ng/dL (reference range, 92-248 ng/dL) or 2.3 nmol/L (reference range, 3.2-8.6 nmol/L). As of the time of manuscript submission, the patient has remained clinically euthyroid.

## Discussion

In the pediatric population, GD is the most common cause of thyrotoxicosis. GD is rare in children younger than 5 years old, and girls are up to 5 times more affected than boys [4]. GD is a clinical syndrome characterized by a hyperthyroid state caused by stimulating TRAb. Our patient demonstrated typical laboratory findings of thyrotoxicosis, which included suppressed TSH, elevated free T4, and elevated total T3. He also had typical symptoms, including tachycardia, hypertension, and exophthalmos. Interestingly, he had negative thyroid stimulating immunoglobulin, TRAb, anti-thyroglobulin, and anti-thyroid peroxidase antibodies, which made the underlying etiology unlikely to be autoimmune.

Thyroid storm is a rare, life-threatening condition in children with thyrotoxicosis. The incidence of TS in children is unclear, and few cases have been reported in the pediatric population to date [2]. Between 2003 and 2014, the incidence of TS in adults ranged from 0.57 to 0.76 cases per 100 000 persons per year in the United States [5]. During an episode of thyrotoxicosis, increased thyroid hormone levels lead to a hypermetabolic state, which causes increased cardiac output, including tachycardia and hypertension [6]. Known precipitating factors of TS include stress from surgical interventions, trauma, infection, and acute iodine load [2].

The diagnosis of TS can be difficult to make in the pediatric population because there is no validated scoring system for this age group. In 1993, Burch and Wartofsky introduced a point scale using precise clinical criteria to identify cases of TS [1]. The criteria include patient temperature, central nervous system (CNS) effects, gastrointestinal-hepatic dysfunction, heart rate, signs of congestive heart failure, signs of atrial fibrillation, and presence of precipitating factors. Even though the scale's validation for use in pediatric patients is unclear, it remains widely used in clinical settings. Our patient had a score of 55 based on the following: tachycardia greater than 140 bpm (25 points), gastrointestinal-hepatic dysfunction with abdominal pain (10 points), CNS disturbance with agitation (10 points), and precipitating event with reported viral bronchitis diagnosed days before admission (10 points), suggesting TS. In 2016, the Japan Thyroid Association and the Japan Endocrine Society published guidelines for the management of TS [7]. Thyrotoxicosis with laboratory confirmation is a prerequisite followed by various symptoms, including CNS manifestations, fever, tachycardia, congestive heart failure, and gastrointestinal/hepatic manifestations. Based on the Japanese criteria, which are not validated for pediatric patients, our patient would have fallen in the definite TS category based on the presence of CNS symptoms (restlessness) and tachycardia along with his laboratory findings of primary hyperthyroidism. Although both scoring systems suggested TS, it is difficult to determine TS in infants based on symptoms of agitation and abdominal pain. However, given the laboratory findings, presenting symptoms, physical examination findings of exophthalmos, and support from the previous criteria, we felt impending TS was the appropriate categorization, thus prompting aggressive treatment.

Paraneoplastic syndromes lead to signs and symptoms affecting multiple organ systems and are associated with malignancies. These disorders are attributed to tumor secretion of hormones, peptides, or cytokines and the immune cross-reaction between malignant and normal tissues [8]. Thyrotoxicosis can be a rare presentation of a paraneoplastic endocrine syndrome. Reported cases have been associated with high levels of HCG in seminomatous and nonseminomatous tumors.

Hepatoblastoma is the most common liver malignancy in children. Most hepatoblastomas present by age 5 years; the highest incidence is in the first 2 years of life. The most common physical examination finding is an abdominal mass. Other nonspecific symptoms are abdominal pain, anorexia, and weight loss. Elevated serum levels of alpha-fetoprotein are seen in up to 90% of cases. Curative treatment consists of surgical resection. However, many patients present with advanced disease stages requiring neoadjuvant chemotherapy before surgical resection [9]. Hepatoblastomas may present with features of paraneoplastic syndrome. Reported features include erythrocytosis, thrombocytosis, hypocalcemia, isosexual precocious puberty, and hypoglycemia [9]. Isosexual precocious puberty is a known manifestation of a pediatric paraneoplastic syndrome caused by hepatoblastoma associated with beta HCG production. Although not reported in the literature in association with hepatoblastoma, hyperthyroidism was previously recognized in patients with nonseminomatous germ-cell tumors that produce beta HCG [10]. Nevertheless, our patient was tested multiple times for elevated serum beta HCG levels, and all tests yielded negative results.

Our patient presented with primary hyperthyroidism, and all common etiologies (autoimmune, thyroid nodule) were ruled out. Although we cannot state with certainty that the hyperthyroidism was a paraneoplastic syndrome, the absence of other causes and the resolution of hyperthyroidism after treatment of the hepatoblastoma led us to believe the hyperthyroidism was in some way related to the diagnosis of hepatoblastoma, and, possibly, a paraneoplastic syndrome.

## Learning Points

- Diagnosing thyroid storm in pediatric patients has many challenges and using multiple diagnostic criteria tools is useful in identifying pending thyroid storm and initiating appropriate therapy.
- Pediatric thyrotoxicosis is most commonly the result of Grave's disease; however, when antibody testing is negative, we must think of uncommon conditions like paraneoplastic syndromes.
- Early identification of uncommon causes of hyperthyroidism is crucial to the diagnosis and treatment of the underlying condition such as a malignancy in our patient.

## Contributors

All authors made individual contributions to authorship. N.V., A.N., S.G., and M.C. were involved in the diagnosis and management of this patient and manuscript submission. All authors reviewed and approved the final draft.

## Data Availability

Data sharing is not applicable to this article as no datasets were generated or analyzed during the current study.

## Abbreviations

CNS: central nervous system
GD: Graves’ disease
HCG: human chorionic gonadotropin
IRB: institutional review board
TRAb: thyrotropin receptor antibody
TS: thyroid storm
